# Association of physical activity, vitamin E levels, and total antioxidant capacity with academic performance and executive functions of adolescents

**DOI:** 10.1186/s12887-019-1528-1

**Published:** 2019-05-17

**Authors:** Ahmad H. Alghadir, Sami A. Gabr, Zaheen A. Iqbal, Einas Al-Eisa

**Affiliations:** 1 Department of Rehabilitation Sciences, College of Applied Medical Sciences, King Saud University, Riyadh, Kingdom of Saudi Arabia; 2 Department of Anatomy, Faculty of Medicine, Mansoura University, Mansoura, Egypt

**Keywords:** Physical activity, Vitamin E, Antioxidant capacity, Academic performance

## Abstract

**Background:**

Although various studies have shown the effect of vigorous physical activity on academic achievements, no studies have investigated the effect of vitamin E levels on academic performance. The present study aimed to assess the association between physical activity, vitamin E levels and total antioxidant capacity on the academic performance and executive functions of adolescents aged 15–18 years.

**Methods:**

The physical activity of participants was assessed according to the time spent engaging in moderate and intense exercise programs. Participants were classified into three groups representing mild, moderate, and high activity. Serum total antioxidant capacity was measured using a colorimetric assay kit. Vitamin E was estimated by the α- and γ-tocopherol levels in fasting serum samples using high-performance liquid chromatography paired with a diode array detector. School grades (ranging from 1.0, very poor; to 10.0, outstanding) were obtained at the end of the academic year to evaluate academic performance and executive functions.

**Results:**

A total of 120 school students (mean age 16.36 ± 0.77 years; 70 boys, 50 girls) participated in the study. Academic performance was higher for students classified as moderately or highly active compared with those in the mild activity group. Serum levels of vitamin E, total antioxidant capacity, and leisure-time physical activity were also higher in the moderate and high activity groups. There was a significant correlation between age, gender, body mind index, α- and γ-tocopherol, total antioxidant capacity, leisure-time physical activity and academic performance.

**Conclusions:**

The academic performance and executive function scores were found to be positively correlated with age, gender, α- and γ-tocopherol, total antioxidant capacity, and physical activity; and were negatively correlated with body mind index. Our findings indicate that physical activity should be promoted during and after school hours, along with a healthy balanced diet including vitamin E.

## Background

Physical activity (PA) has been shown to have various positive effects on bone strength, muscle health, and predisposition to obesity, to name a few [1, 2]. Furthermore, high levels of PA have also been shown to improve cognitive performance, such as memory and learning, for individuals of all ages [3]. However, with the advancement of technology, the time spent by children pursuing physical activities has been overtaken by sedentary activities involving television, computer devices and other screen-based technologies [4].

Regular PA has been shown to augment the antioxidant system and reduce lipid peroxidation [5]. Antioxidants, such as vitamins C and E, selenium, and so on play important roles in protecting the cell membrane from oxidative damage, and supplementation with antioxidants has been shown to enhance physical performance and general health [6–8]. Vitamin E has also been shown to have a role in reducing inflammation and muscle soreness [9, 10], and recent research has suggested that increased intake can help to preserve brain function [11] and protect against nerve cell degeneration [12]. A study in the United States of America (USA) has revealed that a significant number of adults are deficient in vitamin E [13].

Men have been reported to have greater risk of vitamin E deficiency as compared to women in both developed and developing countries [14–16]. A recent study has reported that at least 31% of the USA population is at risk of having at least one vitamin deficiency or anemia, and further shows that it is linked to socio-demographic, life-stage, use of dietary supplement, and dietary adequacy categories [17]. Studies have shown inadequate vitamin E in diets of toddlers aged 18–30 months in Mexico, Kenya, and Egypt [18]. Depletion of vitamin E among children, adolescence, and older populations in developing countries has been related to limited sources of food containing vitamin E and high prevalence of malaria and human immunodeficiency virus in the region [19]. Deficiency of vitamin E has also been associated with low levels of vitamin C, β-carotene and other antioxidants in blood circulation [20]. This supports the theory that deficiency of vitamin E is associated with poor intake and greater oxidative stress [14]. Other factors that have been attributed to vitamin E deficiency include low fat diet, limited inclusion of fruits, vegetables, and whole grains into the diet, and increased consumption of processed food [21, 22]. However, whilst vitamin E deficiency can be deleterious, uncontrolled supplementary intake may lead to toxic effects [23].

Vitamin E has been shown to be of critical importance in early infancy as its deficiency at this stage may predispose severe consequences particularly intraventricular hemorrhage, bronchopulmonary dysplasia and delays in the development of central nervous system [24]. Previous reports have showed that effects of vitamin E were related to both anti-inflammatory and pro-inflammatory properties of both the α-tocopherol and γ-tocopherol isoform which also protects biological cells from oxidative free radical stress via antioxidant enzymes, and increase cytokine production such as Interleukin-2 (IL-2), Interleukin-6 (IL-6), and Tumor Necrosis Factor (TNF) [25–28].

In addition to its role as antioxidant, vitamin E has also shown to be involved in various physiological processes including immune function, control of inflammation, regulation of gene expression and cognitive performance [29, 30]. Vitamin E deficiency was shown to play a role in brain disorders such as cognitive decline and Alzheimer’s disease [31, 32]. Pathological mechanisms affecting motor activity have been shown to be reversed with vitamin E supplementation [33–35].

Physical exercise play a protective role against hippocampal cell injury which produces brain memory loss [36, 37], and facilitates recovery from injury, and improves cognitive function via increase of the expression of many neurotrophic and physiological factors that are involved in neural survival, differentiation, and improvement of memory function [38, 39]. Recently, effects of physical activity on cognitive performance among a healthy older adults were evaluated that show that outcome measures of cognitive performance; in motor praxis, vasomotor organization, thinking operations, and attention and concentration improved significantly following moderate aerobic training for 24 weeks. This has been related to improvement in antioxidant capacity and decline in oxidative stress free radicals [40] Despite these positive effects, parents and teachers often pressurize students to perform better in academia [41], and their inclusion in PA such as physical education, and sports, has received limited support [42].

Although various studies have demonstrated the beneficial effects of vigorous PA on academic achievement; there are, to the best of our knowledge, no studies that correlate the effects of physical activity and vitamin E levels on academic performance. The aim of our study was, therefore, to assess the association between daily physical activity, vitamin E levels, and total antioxidant capacity (TAC) on the academic performance and executive functions of adolescents aged 15–18 years.

## Methods

### Participants

This study was conducted during the October 2014–March 2015 semester at a secondary school. Three hundred students from grade 7 to 9 (aged 15–18 years) from six different senior secondary schools following same academic curricula were invited to participate, of which 220 (73.3%) agreed. They were screened for any health problems, disability, or mental/concentration deficit, and excluded if any such symptom was observed. Finally, 120 participants (70 boys, 50 girls) were included in the study after passing the inclusion and exclusion criteria were applied. During the study period, all students were instructed not to change their normal eating habits.

The demographics and baseline characteristics of the participants are detailed in Table 1. Age, body mass index (BMI), waist to hip ratio (WHR), blood pressure, hemoglobin, and maximum oxygen uptake (VO_2_ max) were measured.

**Table 1:** General characteristics of study participants according to their level of physical activity

	Mild (≤ − 500 MET-min/− week)	Moderate (500–2500 MET-min/− week)	High (≥ − 2500 MET-min/− week)
n	40	50	30
Male/female	25/15	30/20	20/10
Age (years)	16.4 ± 0.78	16.3 ± 0.79	16.4 ± 0.75
BMI (kg/m^2^)	24.9 ± 2.1	23.4 ± 1.45^*^	22.8 ± 1.45^*^
Waist (cm)	85.8 ± 8.9	88.1 ± 10.5	83.6 ± 6.75
Hips (cm)	78.9 ± 5.7	87.04 ± 14.1	99.3 ± 9.12
WHR	1.28 ± 0.14	1.0 ± 0.19^*^	0.84 ± 0.06^*^
Systolic BP (mmHg)	115 ± 2.7	112 ± 1.2	106 ± 6.5
Diastolic BP (mmHg)	82.1 ± 1.9	79.4 ± 3.1	75.4 ± 1.76
Mean HbA1c value (%)	3.1 ± 0.65	2.9 ± 0.51	2.6 ± 0.45
VO_2_ max (ml/kg x min)	38.7 ± 2.4	42.4 ± 3.7^**^	48.7 ± 5.2^**^

Values are expressed as mean ± standard deviation; * *p* < 0.05, ** *p* < 0.01

Key: *METs* metabolic equivalents, *BMI* body mass index, *WHR* waist to Hip ratio, *BP* blood pressure, *VO*_*2*_ *max* maximal oxygen uptake

### Assessment of VO_2_max

Maximum oxygen uptake (VO_2_ max) was evaluated using the ergospirometry on treadmill (inclination of 1%), with initial velocity of 4.5 km/h and increase of 0.5 km/h at each minute until voluntary exhaustion or when one of the following criteria was reached: increase in the VO_2_ lower than 2 ml/kg/Min for the increase in the exercise intensity (plateau); expiratory exchange ratio higher than 1.1; maximum heart rate expected for the age was reached, calculated by the formula (220-age). Prior to the beginning of the test, the individuals performed 3 minutes of warm-up at the 3.1 km/h velocity. Heart rate (HR) was monitored in the electrocardiogram. The respiratory parameters were measured in an open circuit ergospirometric system using the Mix-chamber technique [43–45].

$$ {\mathrm{VO}}_2\ \max\ \left(\mathrm{ml}/\mathrm{kg}\ \mathrm{x}\ \min \right)={\mathrm{VO}}_2\ \left\{220-\mathrm{age}-73-\left(\mathrm{sex}\times \kern0.37em 10\right)/\mathrm{HR}-73-\left(\mathrm{sex}\times \kern0.37em 10\right)\right\} $$

$$ \mathrm{VO}2\ \left(\mathrm{ml}/\mathrm{kg}/\min \right)=\left(1.8\ \mathrm{x}\ \mathrm{work}\ \mathrm{heart}\ \mathrm{rate}\right)/\mathrm{body}\ \mathrm{weight} $$

Sex = 0 for girls and 1 for boys, HR = heart rate at final stage.

### Assessment of physical activity

The PA of the participants was assessed by the time spent performing moderate and intense exercise programs. The activity denoted as leisure-time physical activity (LTPA) was measured by metabolic equivalents (METs), as previously reported [46, 47]. METs were calculated using previously validated questionnaire Global physical activity questionnaire (GPAQ) [48], as MET-minutes/week of the intensity of physical activity according to the formula for computation of MET-minutes/week [49, 50]:

1. {walking MET-minutes/week = × 3.3 walking minutes x walking days}
2. {moderate MET-minutes/week = × 4.0 moderate-intensity activity minutes x moderate days}
3. {vigorous MET-minutes/week = × 8.0 vigorous-intensity activity minutes x vigorous days}
4. {total PA MET-minutes/week = sum of walking + moderate + vigorous MET-minutes/week scores}

The participants were classified into three groups according to PA level; mild (< 500 MET-min/week), moderate (500–2500 MET-min/week) or active (> 2500 MET-min/week). The basal metabolic rate (BMR) and total daily energy expenditure (TEE) were estimated from body mass, height, age, and PA according to the Harris and Benedict equation [51] for obese and non-obese children.

### Blood sampling and analysis

Blood samples were obtained from all the participants in the morning after overnight fasting. Venous blood samples (5 ml) were collected into plain tubes, and the samples were allowed to clot for half an hour following which samples were centrifuged for 15 min at 2000 rpm. Samples were given a coded study identified number and were stored frozen at − 80 °C until analysis.

### Assessment of total antioxidant capacity

Serum TAC was measured using colorimetric assay kit (catalog #K274–100; BioVision Incorporated; CA 95035 USA). The antioxidant equivalent concentrations were measured at 570 nm as a function of Trolox concentration according to the manufacturer’s instructions and calculated using Eq. 1:

$$ \mathrm{Sa}/\mathrm{Sv}=\mathrm{nmol}/\upmu \mathrm{l}\ \mathrm{or}\ \mathrm{mM}\ \mathrm{Trolox}\ \mathrm{equivalent} $$

where Sa is the sample amount (in nmol) read from the standard curve, and Sv is the undiluted sample volume added to the wells.

### Assessment of vitamin E level

Vitamin E levels were estimated from the α- and γ-tocopherol levels measured in fasting serum samples of the participants using high-performance liquid chromatography paired with a diode array detector (Hitachi L-2455; Hitachi Ltd., Tokyo, Japan) (HPLC-DAD). The concentration was calculated by interpolation of results of analysis of α- tocopherol and γ-tocopherol standards (Sigma-Aldrich, Inc., St. Louis, MO, USA) as reported in literature [52]. The inter-assay coefficients of variation were 10.5 and 11.7% for serum α- tocopherol and γ-tocopherol, respectively.

### Assessment of school performance executive function

School grades (ranging from 1.0, very poor, to 10.0, outstanding) of the participants were obtained from the school principals at the end of the academic year. The mean of individual participants’ grades in biology, chemistry, physics, Arabic, English, French, mathematics, social sciences, history, geography, religious studies, physical education and health sciences was taken to represent academic performance. The performance in mathematics alone was reported separately as a measure of executive functioning [53] among participants.

### Statistical analysis

Data were statistically analyzed and expressed as mean ± standard deviation using SPSS 15.0 for Windows (SPSS Inc., Chicago, IL, USA). Comparison of variables was performed using the Mann-Whitney U test and *t*-test parameters. The correlations of PA, serum α- tocopherol and γ-tocopherol with academic performance were examined using Pearson’s correlation coefficient. *P* values < 0.05 were considered to be significant. The correlations of PA, vitamin E and TAC level with academic performance were examined using stepwise linear regression analysis. Variables that have the highest r-squared and strong significance were added in this model. Only, in this study gender, age, BMI, vitamin E forms (α- and γ-tocopherol), and PA scores showed higher r-squared and strong significance. Whereas, VO_2_ max, BMR, TEE, and LTPA showed lower r-squared and deleted from the proposed model.

## Results

The mean age of the participants was 16.36 ± 0.77 years. Classification by PA revealed the following distribution of participants among activity groups mild, 33% (25 male, 15 female); moderate, 42% (30 male, 20 female); and high 25% (20 male, 10 female) (Table 1).

### Comparison between mild, moderate and high activity groups

Compared with the moderate and high activity groups the mild activity group exhibited significantly higher BMI values, WHR, (*p* < 0.05), and lower fitness (VO_2_ max) (*p* < 0.01). On the other hand, there were significantly higher levels of α- and γ-tocopherol, TAC activity and LTPA in the moderate and high activity groups (*p* < 0.05 and *p* < 0.01 respectively) in comparison with the mild activity group (Table 2).

**Table 2:** Association of α- and γ-tocopherol, BMR, TEE rate, and TAC with level of physical activity

	Mild (≤ − 500 MET-min/−week)	Moderate (500–2500 MET-min/−week)	High (≥ − 2500 MET-min/−week)
n	40	50	30
LTPA (METs/week)	62.85 ± 8.3	102.7 ± 11.1^*^	156.5 ± 10.1^**^
BMR (kcal/day)	1.91 ± 0.46	3.5 ± 0.69^*^	3.98 ± 0.61^**^
TEE (kcal/day)	2.4 ± 0.6	3.35 ± 0.76^*^	4.45 ± 0.74^**^
α-tocopherol (mg/L)	3.7 ± 0.7	4.91 ± 0.89^*^	6.31 ± 1.4 ^**^
γ-tocopherol (mg/L)	0.7 ± 0.15	0.91 ± 0.19^*^	1.11 ± 0.24^**^
TAC (nmol /μL)	11.98 ± 3.84	16.45 ± 2.9^*^	31.6 ± 7.3^**^

Values are expressed as mean ± standard deviation; * *p* < 0.05, ** *p* < 0.01

Key: *METs* metabolic equivalents, *LPTA* leisure-time physical activity, *BMR* basal metabolic rate, *TEE* total energy expenditure, *TAC* total antioxidant capacity

Analysis of academic performance and executive function revealed that participants classified into the mild activity group had lower scores of 4.78 ± 0.73 and 4.58 ± 0.68, respectively, compared with the moderate (6.38 ± 0.39, 6.46 ± 0.37) and highly (7.43 ± 0.34, 6.84 ± 0.55) active groups (*p* < 0.01) (Table 3).

**Table 3:** Association of academic performance and physical activity among subjects (*n* = 120)

	Mild (≤ − 500 MET-min/week)	Moderate (500–2500 MET-min/week)	High (≥ − 2500 MET-min/week)
n	40 (33.3%)	50 (41.6%)	30 (25%)
Academic achievement	4.78 ± 0.73	6.38 ± 039^**^	7.43 ± 0.34^**^
Executive function	4.58 ± 0.68	6.46 ± 0.37^**^	6.84 ± 0.55^**^

Values are expressed as mean ± standard deviation; * *p* < 0.05, ** *p* < 0.01

### Correlation between independent variables

Significant correlation was identified between age, gender, BMI, α- and γ-tocopherol, TAC activity, PA level, and academic performance. Academic performance and executive function scores are positively correlated with age, gender, α- and γ-tocopherol, TAC activity and PA score and negatively correlated with BMI (Table 4).

**Table 4:** Stepwise multiple regression analysis academic performance predicted by α- and γ-tocopherol, total antioxidant capacity, body mass index, and physical activity of study participants (*n* = 120)

	Academic achievement		Executive function	
	Β	R2	β	R2
Age	0.025^*^	0.421	0.312^**^	0.751
Gender	0.051^*^		0.055^**^	
BMI (kg/m^2^)	−0.041^*^		−0.035^**^	
α-tocopherol (mg/L)	0.071^*^		0.085^**^	
γ-tocopherol (mg/L)	0.021^*^		0.028^**^	
TAC (nmol /μL)	0.048^*^		0.065^**^	
Physical activity score	0.092^*^		0.049^**^	

Estimated standardized regression coefficients (β) and variance explained (R^2^) are presented, * *p* < 0.05; ** *p* < 0.01

## Discussion

This study demonstrates the association of different levels of PA, vitamin E levels, and TAC with the academic performance and executive functions among adolescents aged 15–18 years. We found that BMI and WHR were significantly higher in participants who were classified as having mild activity levels, while fitness and achievement scores with respect to academic performance and executive function were lower in comparison with the moderate and highly active groups. On the other hand, significantly higher levels of α- and γ-tocopherol, TAC activity, and LTPA were observed in participants in the moderate and high activity groups. Academic performance and executive function scores were found to be positively correlated with age, gender, α- and γ-tocopherol, TAC activity, and PA score; and negatively correlated with BMI.

Various studies have reported the possible correlation between PA and academic achievements. Some of studies report randomized controlled trials, and most of them focus only on primary school students [54]. Most of these studies include only vigorous activities [55] and do not consider mild and moderate PA. This study included all types of PA that involve increased energy expenditure; including exercise, sport activities and activities that comprise school physical education curricula. Furthermore, previous studies neglected to include any investigation of nutrition status of children. To the best of our knowledge, this is the first study to consider serum antioxidant levels while correlating PA and academic performance.

There is a paucity of research into the relationship between PA and academic performance based on ethnicity [1]. The present study was conducted at a secondary school in Middle East, where no such studies have been carried out previously. However, sedentary behavior, including increased time spent sitting while watching TV and using computer or similar devices, has been linked to poor physical fitness, lower energy expenditure, and higher BMI leading to obesity in this region [4]. Our results showing significantly higher BMI and WHR values and lower fitness in the mild activity group (more sedentary and low intensity physical activities) further support this study. Encouraging PA in and after school could promote fitness along with decreased predisposition to obesity, and consequent improved academic performance.

Past studies have reported the association between school time PA and academic performance through various direct and indirect mechanisms including physiological, psychological, cognitive, emotional, and learning mechanisms [56, 57]. PA has been shown to increase cerebral blood flow and perfusion of motor areas of the brain [58, 59]. It also increases brain neural activity and arousal [60] resulting in increased cerebral serotonin levels, which are reported to have a calming effect on body [61]. Increased synaptic transmission, neurotrophin concentration, and neurogenesis; and decreased free radicals following PA have been shown to facilitate the memory process [62, 63].

While published reviews of the literature have revealed that relationship between PA and academic performance usually presents as positive correlation or no correlation [64, 65], there are some studies that suggest a negative correlation between these variables [56]. This difference in results may arise due to the specific varieties of PA and academic performance outcomes that were evaluated in the different studies. The majority of these studies did not include PA other than those included in the school curricula. To overcome this, our study included evaluation of PA that students engaged in outside of school hours. A recent survey of the PA, diet habits and TV habits of students in an Arab region [2], revealed that the majority (71%) of respondents reported they “eat and study” during school breaks rather than “eat and play”. Lunch breaks or recess periods are provided to students in school to take a break from study and rejuvenate themselves. Teachers and school authorities should make sure that students engage in some form of PA during this period.

Research has shown that vitamin E helps to preserve brain function and protect against nerve cell degeneration [11, 12]. This in turn contributes to the preservation of cognitive function and promotes the learning process. Our results reflect this as a positive correlation between serum vitamin E levels and overall academic performance. It has been shown that detection of neuropsychological and physiological deficits early in a child’s development can predict poor academic performance [66]. Therefore, the serum levels of vitamin E and other antioxidants should be included in such an assessment in order to develop a rehabilitative plan for children with such defects.

Serum levels of TAC were estimated among all participants of this study and it was shown to be significantly higher in those with moderate and high PA compared to those with mild PA. This increment in TAC was also shown to be closely related to higher levels of α- and γ-tocopherol as isoforms of vitamin E, and LTPA. Additionally, academic performance and executive function scores were also correlated positively with α- and γ-tocopherol, TAC activity, PA score. Previous reports show that potential effects of vitamin E are related to both anti-inflammatory and pro-inflammatory properties of both the α- and γ-tocopherol isoforms which protect biological cells from oxidative free radical stress via producing antioxidant enzymes, and increase cytokine production such as IL-2, IL-6, and TNF [25–28, 67]. Thus, populations with controlled food containing vitamin E isoforms (α- and γ-tocopherol) were safe from severe consequences such as elevated oxidative stress, greater cellular inflammations, and poor cognitive performance [19, 68].

PA has been shown to play an important role in improving cognitive functions among older adults by modulating redox and inflammatory status, and consequently increasing the TAC activity, and reducing oxidative free radical stress parameters such as MDA, 8-OHdG, etc. [40] Vitamin E is a lipid soluble antioxidant that scavenges free radicals to protect cell membranes and lipoproteins from oxidative damage and significantly increases TAC in physically active people [69, 70]. Vitamin E has been shown to be increased in lymphocytes of the participants following endurance exercises [71], which further supports the correlation between PA, vitamin E levels, and TAC activity as seen in our study.

Children who face difficulties in learning may benefit from the provision of a balanced diet including vitamin E and other antioxidants. Breakfast has been suggested to be the most important meal of the day [72], and the regular intake of a healthy and timely breakfast has been shown to improve cognitive and memory functions of the brain [73], as well as decreasing the likelihood of weight gain [74]. Many children indicate that they miss breakfast either due to a dislike for the offered foods, or for time pressure [2]. With the availability of fast food and carbonated drinks, in school cafeterias, children often do not consume the recommended quantities of healthy foods, including milk [74]. Furthermore, consumption of processed food has been shown to be a major cause of vitamin E deficiency [13].

Although participants of this study were instructed to have their normal diet during data collection, correlation between nutrition, executive function, and academic performance could not be measured. Self-reported questionnaire was used to measure physical activity among participants. To further elucidate the correlation of vitamin E and PA, especially in relation to age and gender, a supervised aerobic training model is recommended for further studies.

## Conclusions

Groups of adolescents who were moderately of highly active were found to have significantly higher levels of vitamin E, TAC activity, LTPA, and academic performance compared with mildly active participants. The academic performance and executive function scores were found to be positively correlated with age, gender, α- and γ-tocopherol, TAC activity, and PA score; and negatively correlated with BMI. Vitamin E levels and TAC activity could be used as indicators for improving executive function, PA and academic performance among students.

## Abbreviations

BMI: Body mass index
BMR: Basal metabolic rate
GPAQ: Global physical activity questionnaire
HR: Heart rate
IL-2: Interleukin-2
IL-6: Interleukin-6
LTPA: Leisure-time physical activity
MET: Metabolic equivalents
PA: Physical activity
TAC: Total antioxidant capacity
TDEE: Total daily energy expenditure
TNF: Tumor Necrosis Factor
USA: United States of America
VO_2_ max: Maximum oxygen uptake
WHR: Waist to hip ratio
